# Clinical and ultrasonographic features preceding a difficult-to-treat phenotype in psoriatic arthritis: a retrospective real-world exploratory cohort study

**DOI:** 10.3389/fimmu.2026.1869052

**Published:** 2026-08-26

**Authors:** Elisa Bellis, Mariele Gatto, Claudio Cruciani, Federico Favaro, Eleonora Croce, Francesca Luppi, Federica Agugliaro, Claudia Garulli, Federica Laterza, Gloria Crepaldi, Valeria Data, Claudia Lomater, Marta Saracco, Annamaria Iagnocco

**Affiliations:** 1 Academic Rheumatology Centre, Department of Clinical and Biological Sciences, University of Turin, Mauriziano Hospital, Turin, Italy; 2 Department of Translational Medicine, Università del Piemonte Orientale UPO, Novara, Italy

**Keywords:** difficult-to-treat, predictors, psoriatic arthritis, real-world, ultrasound

## Abstract

**Objectives:**

To explore clinical and ultrasonographic features preceding the development of a difficult-to-treat (D2T) phenotype in a real-world cohort of patients with psoriatic arthritis (PsA).

**Materials and methods:**

In this retrospective cohort study, consecutive PsA patients (January 2020-September 2024) were included. D2T PsA was defined according to a pragmatic operational definition based on persistent disease activity despite ≥2 biologic or targeted synthetic disease-modifying antirheumatic drugs (b/tsDMARDs). Baseline demographic and clinical data were collected. Ultrasound (US) assessment included synovitis, erosions, tenosynovitis, enthesitis, bursitis, and dactylitis, using approved definitions and scoring systems. Predictors of D2T were analyzed using Cox regression models.

**Results:**

A total of 152 patients were included (81 D2T, 71 non-D2T), with US data available in 117. D2T patients were younger at diagnosis (47.3 ± 11.4 vs 53.2 ± 10.1 years, p<0.001). In the overall multivariable Cox model, no baseline clinical variable independently predicted D2T. However, when diagnostic delay was categorized using a ≥1-year threshold, Kaplan-Meier analysis showed reduced D2T-free survival in patients with diagnostic delay ≥1 year (log-rank p=0.025). When analyses were stratified according to predominant disease pattern, diagnostic delay ≥1 year remained independently associated with D2T in both peripheral [HR 1.74 (95% CI 1.05–2.88), p=0.032] and axial PsA [HR 3.18 (1.22–8.27), p=0.018]. In axial disease, fibromyalgia [HR 2.45 (1.12–5.36), p=0.025] and enthesitis [HR 2.49 (1.08–5.70), p=0.033] were additional predictors. In multivariable Cox regression, higher mean bilateral Global OMERACT/EULAR US Synovitis Score (GLOESS) at the first metatarsophalangeal joint (MTP) was directly associated [HR 3.21 (1.47–6.99), p=0.003] while mean GLOESS at the wrist was inversely [HR 0.52 (0.33–0.80), p=0.003] associated with D2T.

**Conclusions:**

In this retrospective exploratory cohort, diagnostic delay of at least one year and selected US features were associated with later D2T PsA classification. First MTP GLOESS was positively associated with D2T classification, whereas wrist GLOESS showed an inverse association. These findings should be interpreted cautiously because of the retrospective design, variable US timing, and need for external validation.

## 1 Introduction

Psoriatic arthritis (PsA) is a chronic inflammatory disease with a highly heterogeneous clinical presentation, encompassing musculoskeletal, cutaneous, and systemic manifestations (1). Despite therapeutic advances, up to 20% of patients (2) continue to experience persistent disease activity and multiple treatment failures, a condition increasingly referred to as difficult-to-treat (D2T) PsA. This concept derives from D2T rheumatoid arthritis (RA) but requires a specific framework to reflect the clinical complexity of PsA (1).

Recently, EULAR proposed new consensus definitions, distinguishing the umbrella construct of difficult-to-manage PsA from the more stringent treatment-refractory PsA. The former includes patients with persistent signs and symptoms of disease despite multiple advanced treatments, regardless of whether these manifestations are driven by active inflammation, whereas the latter identifies the subgroup of patients with persistent objective inflammatory activity despite appropriate treatment (3). Similarly, Group for Research and Assessment of Psoriasis and Psoriatic Arthritis (GRAPPA) has recently distinguished the broader concept of complex-to-manage PsA from treatment-refractory PsA, the latter referring to a subgroup of non-responder patients with evidence of active inflammation despite multiple therapeutic failures (4).

Nevertheless, previous studies investigating D2T PsA have used heterogeneous definitions, reflecting the complexity of this clinical phenotype. Proposed D2T PsA criteria encompass (i) failure of at least two biologic or targeted synthetic disease-modifying antirheumatic drugs (b/tsDMARDs) with different mechanisms of action; (ii) persistent activity in at least one PsA domain; (iii) comorbidities influencing disease management; and (iv) discrepancy between patient-reported symptoms and objective inflammatory findings (5).

Disease refractoriness may be driven by comorbidities and mental health disorders (6), heterogeneous domain involvement (1), and discordance between subjective and objective measures (7). Identifying patients at risk of developing D2T PsA is therefore crucial.

Musculoskeletal ultrasound (US) has become an increasingly valuable tool in the diagnosis and management of PsA, owing to its cost-effectiveness and its ability to detect both inflammatory and structural abnormalities. US can support the differential diagnosis with other inflammatory arthritides and may help distinguish inflammatory disease activity from non-inflammatory conditions that can mimic or coexist with PsA, such as osteoarthritis and fibromyalgia (8).

Despite its established role in clinical practice and its widespread use in studies investigating patients with psoriasis at risk of developing PsA during the preclinical phase of the disease (8), US has not yet been adequately explored as a tool for the early identification of patients at risk of developing D2T PsA.

While several studies have explored clinical and imaging predictors (2, 5, 9–14), to our knowledge no data specifically address whether US features preceding multiple biologic failures characterize patients evolving toward a D2T phenotype.

Accordingly, this study aimed to explore clinical and US factors associated with D2T classification in a real-world PsA cohort.

## 2 Materials and methods

### 2.1 Patient cohort

In this retrospective study, we analyzed consecutive patients with PsA followed at a tertiary referral rheumatology center (Academic Rheumatology Centre, Mauriziano Umberto I Hospital and University of Turin, Italy) from January 2020 to September 2024. Given the retrospective design of the study, no formal sample size calculation was performed and consecutive inclusion was adopted to reduce selection bias.

Patients were eligible for inclusion if they were 18 years or older and met the CASPAR classification criteria for PsA (15). Exclusion criteria included incomplete medical records, a follow-up period of less than six months by March 2025, and the inability to provide informed consent.

Patients’ medical records were reviewed to collect demographic and clinical data, including age, sex, smoking status, body mass index (BMI), comorbidities, including fibromyalgia, defined according to the AAPT Diagnostic Criteria (16), disease duration at inclusion, time from symptoms to PsA diagnosis, and both previous and ongoing therapies. The pattern of PsA involvement was also recorded, distinguishing between peripheral and axial involvement, with axial disease defined according to the Assessment of SpondyloArthritis international Society (ASAS) classification criteria for axial spondyloarthritis (17). For stratified analyses, patients were classified according to the predominant phenotype documented during follow-up prior to D2T classification in D2T patients and prior to the last available follow-up visit in non-D2T patients; these subgroups were not mutually exclusive, as patients with a prevalent phenotype could also present concomitant involvement in the other domain and were therefore included in both analyses. Imaging findings were considered when available as part of routine clinical evaluation.

D2T PsA was defined as failure of at least two b/tsDMARDs with different mechanisms of action, in the presence of persistent clinical disease activity assessed by the treating rheumatologist and documented in the medical records. The D2T definition adopted in this study reflects the operational definition used for this retrospective analysis and the data available in a real-world setting. This pragmatic approach was intended to identify patients with characteristics broadly consistent with, although not fully reflective of, the EULAR difficult-to-manage construct (3).

The date of D2T classification was defined as the first clinical visit at which failure of at least two b/tsDMARDs with different mechanisms of action and persistent disease activity were simultaneously documented. When available, Disease Activity Index for Psoriatic Arthritis (DAPSA) (18, 19) scores were used to support the assessment of persistent disease activity. In the absence of DAPSA, persistent disease activity was defined according to the treating rheumatologist’s assessment recorded in the medical chart.

### 2.2 US assessment

Joint US was performed at baseline and during routine clinical follow-up. Only US encompassing extensive articular mapping were considered for the analysis. Eligible US examinations assessed the following joint sites: wrists, metacarpophalangeal joints (MCP), hand proximal interphalangeal joints (PIP), knees, ankles and metatarsophalangeal joints (MTP). The lateral epicondyles of the humerus, medial femoral condyles, Achilles tendon insertions, and plantar fascia insertions were routinely assessed bilaterally, while additional entheseal sites were evaluated according to patients’ symptoms and clinical presentation. In patients who subsequently developed D2T PsA, the last US examination performed before fulfilment of D2T criteria was considered, while in non-D2T patients, the most recent available US during follow-up was taken into account. Because of the retrospective real-world design, US examinations were performed according to routine clinical practice rather than a predefined research protocol. Therefore, the timing of US assessments in relation to treatment milestones and D2T classification was not standardized.

All US assessments were performed by two certified expert rheumatologists from our center following a standardized scanning technique (20), and using an Esaote MyLabX8 US machine equipped with high-resolution linear probes (4–15 MHz and 8–24 MHz), reflecting real-world clinical practice. US examinations included standardized patient positioning, probe placement, and multiplanar scanning according to EULAR recommendations (20). Power Doppler settings were optimized for the detection of inflammatory signals following routine procedures adopted in our US unit.

US examinations were performed as part of routine clinical care. Therefore, sonographers were not blinded to patients’ clinical status or treatment history, whereas future D2T classification was unknown at the time of image acquisition. Inter-reader reliability was not formally tested due to the retrospective design. All areas were evaluated for synovitis, erosions, tenosynovitis, enthesitis and bursitis, according to international definitions of pathology (21–24).

Synovitis was assessed according to the EULAR-OMERACT consensus definitions and quantified using the Global OMERACT/EULAR Ultrasound Synovitis Score (GLOESS) (22, 25, 26). Although originally developed in RA, GLOESS has been used in PsA US studies and clinical trials (26, 27), where it provided a standardized approach for the assessment of synovitis, and was therefore adopted in the present study. Enthesitis was evaluated according to the OMERACT consensus definition and scoring system (24). Tenosynovitis, erosions and bursitis were assessed according to internationally accepted OMERACT definitions of US pathology (21), while dactylitis was evaluated according to the DACTylitis glObal Sonographic (DACTOS) score when clinically evident (18, 23). Potential predictors of D2T phenotype across baseline clinical and US findings were assessed.

### 2.3 Ethics

The study received approval from the local Institutional Ethics Committee (Interagency Territorial Ethics Committee A.O.U. Città della Salute e della Scienza, No. 139/2023). The approved study protocol included retrospective review of both clinical records and US examinations collected during routine clinical care. All participating patients provided written informed consent.

### 2.4 Statistics

Continuous variables were reported as mean (standard deviation) or median (interquartile range), as appropriate. Categorical variables were expressed as frequencies and percentages. Between-group comparisons were performed using t-test or Mann-Whitney U test for continuous variables and χ² test for categorical variables. To investigate predictors of D2T PsA, two separate multivariable Cox proportional hazards regression models were developed: one including baseline clinical variables and one including ultrasonographic variables.

Time-to-event was calculated from PsA diagnosis to D2T classification or last follow-up. Clinical covariates were selected based on clinical relevance and prior literature. Ultrasonographic variables were initially explored descriptively; in order to limit model complexity and reduce the risk of overfitting, variables showing a signal of association (p<0.10) and/or deemed clinically relevant were then selected for univariable and multivariable Cox analyses. Given the exploratory nature of the ultrasonographic analyses and the limited number of events, a backward stepwise Cox approach was was used as an exploratory variable-selection approach. The candidate variables included PIP synovitis, mean bilateral GLOESS at the first MTP and wrist joints, and extensor carpi tenosynovitis grade.

Models were adjusted for age, sex, body mass index, while differences in follow-up time were accounted for through the time-to-event structure of the Cox model. Proportional hazards assumptions were assessed graphically using log-minus-log survival plots. Hazard ratios (HR) with 95% confidence intervals (CI) were calculated. Kaplan-Meier survival curves were generated for key predictors and compared using the log-rank test. Analyses were performed on available data and no imputation procedures were applied. Statistical significance was set at p < 0.05 (two-sided). Analyses were performed using IBM SPSS version 29.0.1.0.

## 3 Results

Out of 178 patients screened, 152 fulfilled CASPAR criteria for PsA and were included in the clinical analysis. Twenty-six patients were excluded because they did not meet PsA classification criteria. Among them, 81 were classified as D2T PsA and 71 as non-D2T PsA (**Supplementary Figure 1**). Patients in the D2T group met D2T criteria after a median (IQR) of 5.38 (3.51-9.83) years from PsA diagnosis. The overall duration of follow-up was comparable between groups, with a median (IQR) of 8.19 (5.72-13.21) years in the D2T group and 7.42 (5.42-10.33) years in the non-D2T group (p = 0.213). The lag time between symptom onset and PsA diagnosis was also similar between groups.

Patients classified as D2T PsA were younger at diagnosis. No significant between-groups differences were observed in terms of presence of psoriasis, fibromyalgia, or prevalent disease pattern (axial or peripheral). Nail involvement was more frequently observed in the D2T group (60.4% vs. 39.4%), although this difference did not reach statistical significance.

Clinical and demographic characteristics of both groups are summarized in **Table 1**.

**Table 1:** Baseline clinical and demographic characteristics of PsA cohort.

Clinical and demographic characteristics	D2T PsA n=81	Non-D2T PsA n=71	p-value
Gender (female), n (%)	42 (51.8)	35 (49.2)	0.751
Age at diagnosis, years, mean (± SD)	47.3 ± 11.4	53.2 ± 10.1	<0.001
BMI, kg/m² mean (± SD)	30.5 ± 22.3	31.1 ± 31.4	0.121
Time from symptoms to PsA diagnosis, years, median (IQR)	1.17 (0.0-6.0)	0.94 (0.1-4.09)	0.998
Time to first bDMARDs, years, median (IQR)	1.89 (0.17-4.76)	1.54 (0.34-3.11)	0.431
Comorbidities, n (%)	44 (54.3)	32 (45.1)	0.415
Fibromyalgia, n (%)	49 (60.4)	28 (39.4)	0.181
Smoking status, n (%)	48 (59.3)	29 (40.8)	0.277
Predominant disease pattern			
Peripheral, n (%)	45 (55.5)	32 (45.1)	0.181
Axial, n (%)	44 (54.3)	33 (46.4)	0.548
Enthesitis, n (%)	41 (50.6)	36 (50.7)	0.648
Psoriasis, n (%)	44 (54.3)	32 (45.1)	0.184
Nail involvement, n (%)	49 (60.4)	28 (39.4)	0.209
TJC	8.78 (8.85)	5.97 (6.00)	<0.001
SJC	0.88 (1.46)	0.33 (0.85)	<0.001

PsA, psoriatic arthritis; D2T, difficult-to-treat; Non-D2T, non-difficult-to-treat; SD, standard deviation; IQR, Interquartile Range; BMI, Body Mass Index; bDMARDs, biological disease-modifying anti-rheumatic drugs; TJC tender joint count; SJC swollen joint count.

Previous b/tsDMARDs treatment throughout the clinical history of both groups is summarized in **Supplementary Table 1**.

### 3.1 Clinical predictors of D2T PsA

**Figure 1:**
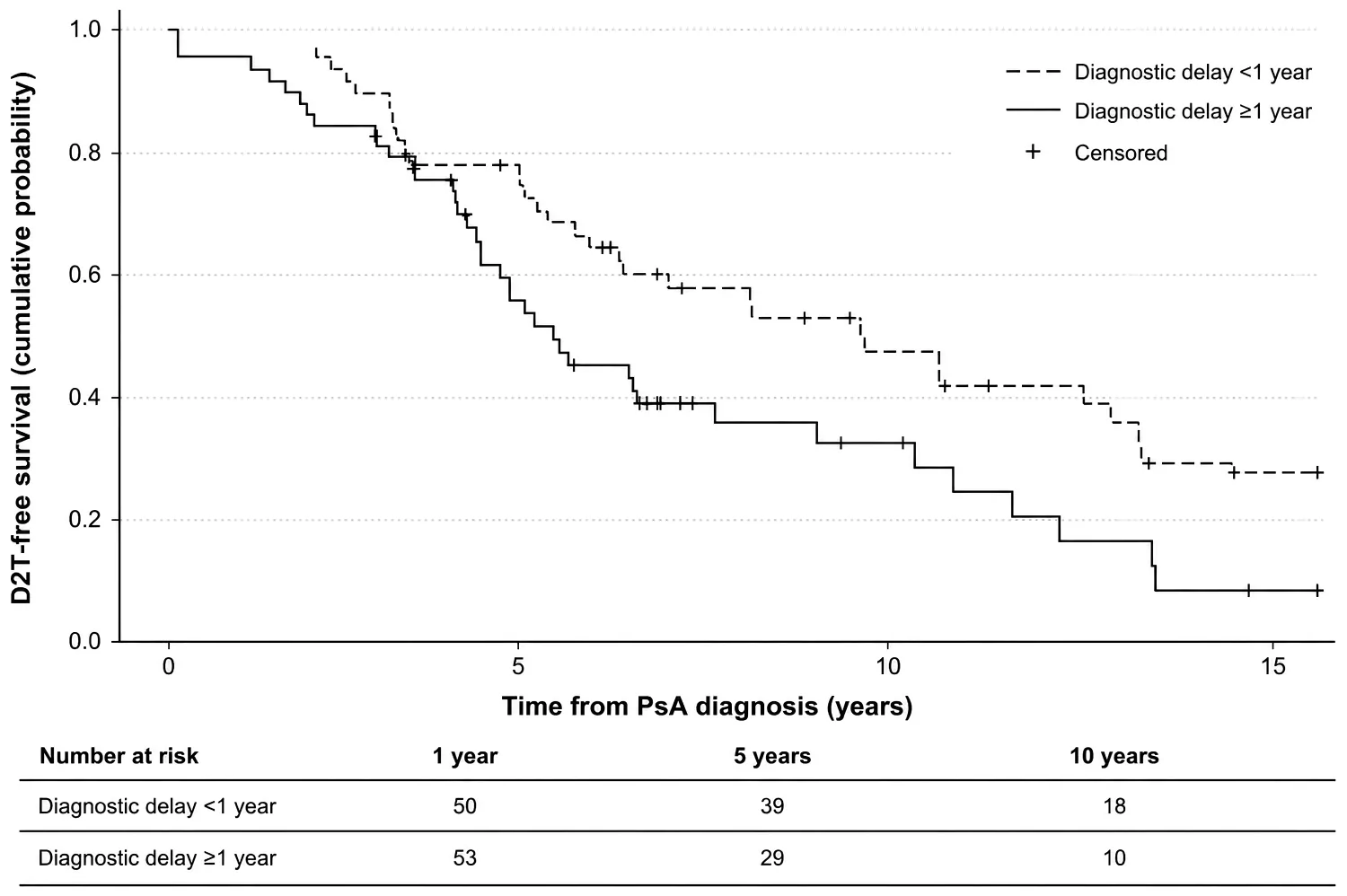
Kaplan-Meier curves for D2T-free survival stratified by diagnostic delay (≥1 year vs <1 year). D2T-free survival was compared between patients with diagnostic delay ≥1 year and <1 year using the log-rank (Mantel-Cox) test (p = 0.025). Numbers at risk are shown below the graph. D2T, difficult-to-treat.

Considering the whole cohort, in multivariable Cox regression analysis including disease pattern, nail involvement, concomitant fibromyalgia, smoking status, and diagnostic delay, no significant predictors of D2T classification emerged, despite an association with enthesitis at univariable analysis (**Table 2**). However, when diagnostic delay was categorized using a ≥1-year threshold, Kaplan-Meier analysis demonstrated a separation of D2T-free survival curves (**Figure 1**). Patients with a diagnostic delay ≥1 year showed an earlier decline in D2T-free survival compared to those diagnosed within 1 year. The difference between groups was statistically significant according to the log-rank test (p = 0.025). Sensitivity analyses using alternative diagnostic delay thresholds showed a similar direction of association for delays ≥2 years, whereas a 6-month threshold was not significantly associated with D2T classification (data not shown).

**Table 2:** Univariable and multivariable Cox regression analysis of clinical predictors of D2T PsA (overall cohort).

Variable	HR univariate (95% CI)	p-value	HR multivariate (95% CI)	p-value
Diagnostic delay	1.437 (0.909–2.273)	0.121	1.021 (0.939–1.110)	0.632
Fibromyalgia	1.433 (0.895–2.296)	0.134	0.344 (0.058–2.040)	0.240
Smoking status	1.513 (0.901–2.540)	0.117	0.799 (0.103–6.192)	0.830
Nail involvement	1.142 (0.709–1.839)	0.585	0.877 (0.229–3.357)	0.848
Peripheral pattern	1.144 (0.356–3.680)	0.822	0.785 (0.012–52.10)	0.910
Axial pattern	0.941 (0.593–1.493)	0.796	0.502 (0.088–2.864)	0.438
Enthesitis	1.694 (1.063–2.697)	0.026	0.938 (0.256–3.445)	0.924

Variables included in the model: disease pattern, nail involvement, fibromyalgia, smoking status, lag time to diagnosis. Model adjusted for baseline age, gender, body mass index, follow-up duration. D2T, difficult-to-treat; HR hazard ratio; CI confidence interval.

When analyses were stratified according to predominant peripheral or axial disease pattern, diagnostic delay ≥1 year remained independently associated with D2T classification in both peripheral (HR 1.74, 95% CI 1.05–2.88), p=0.032) and axial subgroups (HR 3.18, 95% CI 1.22-8.27, p=0.018). In the axial subgroup, fibromyalgia (HR 2.45, 95% CI 1.12-5.36, p=0.025) and enthesitis (HR 2.49, 95% CI 1.08–5.73, p=0.033) were also independently associated with D2T classification (**Table 3**).

**Table 3:** Clinical predictors of difficult-to-treat psoriatic arthritis (multivariable Cox regression) in predominantly peripheral and axial involvement subgroups.

Peripheral involvement subgroup (n = 143)			
Variable	HR	95% CI	p-value
Diagnostic delay ≥1 year	1.74	1.05–2.88	0.032
Axial involvement subgroup (n = 61)			
Variable	HR	95% CI	p-value
Diagnostic delay ≥1 year	3.18	1.22-8.27	0.018
Fibromyalgia	2.45	1.12-5.36	0.025
Enthesitis	2.49	1.08-5.73	0.033

Models adjusted for age at diagnosis, sex, body mass index and smoking status. HR, hazard ratio.

### 3.2 Ultrasonographic predictors of D2T PsA

Among the 152 PsA patients, 117 had at least one available US examination and were included in the US analysis (55 D2T PsA, 62 nD2T PsA). Time elapsed from PsA diagnosis to examined US assessment was comparable between groups (D2T vs. non-D2T patients: 3.84 (2.27-4.88) vs. 3.45 (2.68-4.91) years, p=0.976). Among D2T patients, the median (IQR) interval between US assessment and D2T classification was 2.18 (1.33-4.20) years.

PIP synovitis was more frequently observed in patients subsequently classified as D2T PsA (n=6, 10.9% vs. n=1, 1.6%, p=0.045). No significant differences were observed between groups regarding synovitis at other joint sites (**Supplementary Table 2**). Similarly, no significant differences between groups were observed in tenosynovitis, erosions, enthesitis, bursitis, or dactylitis at the selected US examination. (**Supplementary Table 2**).

MTP synovitis was observed in n=8 (14.5%) D2T patients and n=5 (8.1%) non-D2T patients. The GLOESS score at the level of the first MTP joint (bilaterally) was significantly higher in patients who later developed D2T PsA (mean (SD): 0.10 (0.33) vs. 0, p=0.022). No correlation was found between GLOESS and DAPSA scores in the subset of patients with available DAPSA assessment (n=23; **Supplementary Table 3**). The grade of tenosynovitis at the level of the extensor carpi tendons was higher in patients who subsequently developed D2T PsA (mean (SD): 0.11 (0.33) vs. 0.02 (0.14), p = 0.054). Extensor carpi tenosynovitis was observed in n=6 (10.9%) D2T patients and n=2 (3.2%) non-D2T patients.

In multivariable stepwise Cox regression analysis, higher mean bilateral GLOESS at the first MTP joint was independently associated with D2T classification (HR 3.21, 95% CI 1.47–6.99, p=0.003), while higher mean GLOESS at the wrist showed an inverse association (HR 0.52, 95% CI 0.33–0.80, p=0.003) (**Table 4**). Wrist synovitis was observed in n=27 (49.0%) D2T patients and n=30 (48.4%) non-D2T patients, respectively, (p=0.990).

**Table 4:** Ultrasound (US) predictors of difficult-to-treat psoriatic arthritis (Cox multivariable hazard model adjusted for baseline confounders and lag time between diagnosis and joint US).

Variable	HR univariate (95% CI)	p-value	HR multivariate (95% CI)	p-value
PIP synovitis	2.03 (0.87-4.76)	0.103	1.95 (0.75-5.08)	0.171
Extensor carpi tenosynovitis	2.05 (0.87-4.81)	0.100	1.43 (0.58-3.54)	0.437
**Mean bilateral GLOESS first MTP (bilateral)**	**3.14 (1.43-6.89)**	**0.004**	**3.21 (1.47-6.99)**	**0.003**
**Mean GLOESS wrist**	**0.63 (0.31-1.28)**	**0.197**	**0.52 (0.33-0.80)**	**0.003**

Multivariable Cox regression using backward stepwise selection; adjusted for age, sex, BMI and time from diagnosis to US examination. HR, hazard ratio; PIP, Proximal Interphalangeal; GLOESS, Global OMERACT/EULAR US Synovitis Score; MTP, Metatarsophalangeal.Bold values indicate statistically significant results in the multivariable analysis.

## 4 Discussion

The expansion of the therapeutic armamentarium in inflammatory arthropathies over the past two decades has profoundly transformed the management of PsA (28), making complete remission a realistic treatment target, while introducing the concept of treatment resistance in patients failing multiple therapeutic lines.

Over the years, terms such as “refractory”, “D2T” or “complex” PsA have been adopted by different authors using different definitions (29), and lately a consensus to establish standardized definitions for difficult-to-treat and treatment-refractory PsA has been released, distinguishing persistent inflammatory disease from multifactorial drivers of ongoing symptoms despite appropriate therapy. In this regard, the need to identify early predictors of treatment resistance is becoming increasingly relevant (4).

In the current work, we adapted the RA-related definition of D2T to our large real-world cohort of PsA patients and assessed both clinical and US predictors of D2T PsA, which may aid earlier patient stratification and treatment.

D2T PsA patients were defined as having failed 2 or more targeted drugs with different mechanisms of action. Across our whole cohort, a diagnostic delay ≥1 year identified a clinically meaningful separation in D2T-free survival and, although not significant as a continuous variable in the overall multivariable model, remained independently associated with D2T classification in stratified analyses. This observation is in keeping with previous studies showing that diagnostic delay is associated with worse radiographic and functional outcomes, higher rates of DMARDs failure (30, 31), and a lower likelihood of achieving minimal disease activity (32, 33). Yolbaş et al. also reported a higher rate of musculoskeletal surgeries during the pre-diagnostic phase of PsA compared with the period following diagnosis, further emphasizing the importance of timely diagnosis in preventing structural damage and disability (34). Our real-world findings therefore reinforce the critical importance of early diagnosis to prevent the evolution toward a refractory disease phenotype and the importance of elucidating the stages preceding the onset of PsA, with the 2023 European Alliance of Associations for Rheumatology (EULAR) definitions of “Pre-PsA” providing rational framework for future studies in this direction (35).

No specific joint pattern was identified as being more strongly associated with diagnostic delay in our study, despite previous data (33) reporting a longer lag time to diagnosis in patients with enthesitic involvement, whereas those presenting with polyarthritis or dactylitis experienced a shorter delay. In this regard, we did not identify a predominant baseline pattern of joint involvement among patients who later developed D2T PsA; however, enthesitis emerged as a critical driver of D2T phenotype within a predominant axial involvement. Evidence on this topic remains conflicting. Vassilakis et al. (13) and Philippoteaux et al. (12) reported an increased D2T risk associated with axial involvement, whereas Alp et al. (2) observed a higher risk in patients with a peripheral phenotype. Finally, data from the Rheumatic Diseases Portuguese Registry demonstrated that both polyarticular disease and enthesitis were associated with the subsequent development of D2T PsA (9). The current inconsistencies across studies emphasize the complexity of D2T PsA in terms of affected sites and underscore the need for further studies to determine whether specific joint patterns confer an increased risk, provided that the addition of multiple inflammatory joint sites likely supports refractoriness.

Focusing the analysis on patients with axial PsA, fibromyalgia was additionally associated with a greater risk of treatment failure. Fibromyalgia affects approximately 18–25% of PsA patients (36) and has previously been suggested as a potential risk factor for developing D2T PsA (2, 11), as it negatively impacts disease activity, functional indices, and drug retention rate (6, 37, 38), potentially leading to misinterpretation of disease activity and inappropriate treatment adjustments (36). This aspect is particularly relevant when interpreting D2T classification, as fibromyalgia-related symptoms may contribute to repeated treatment changes and fulfilment of D2T criteria as a result of non-inflammatory symptom burden rather than truly treatment-refractory inflammatory PsA.

Joint US performed at a pre-D2T stage added novel prognostic information in our cohort. Hand PIP synovitis, high first MTP GLOESS scores, and more severe carpal tenosynovitis were associated with risk of D2T PsA, although the absolute numbers of patients displaying overt alterations was low, likely due to most included US being performed in the early phases of disease. Moreover, these findings suggest a potential relevance of articular features that are often overlooked in conventional disease activity indices (such as tenosynovitis and first MTP) in contributing to disease refractoriness. Nevertheless, the absolute burden of first MTP synovitis was low, and the clinical relevance of this association should therefore be interpreted cautiously and considered hypothesis-generating pending external validation.

Notably, US variables expressed on a continuous scale (e.g., GLOESS, tenosynovitis grading) may offer greater predictive value than dichotomous findings, as they capture the full spectrum of inflammatory severity.

Severity of wrist synovitis, in particular, predicted a more favorable course in our cohort, consistent with trial data on secukinumab, where wrist synovitis was reported as one of the most treatment-responsive sites (27). This finding may indicate that certain inflammatory patterns are more treatment-responsive and therefore less closely linked to the development of cumulative refractoriness. However, this interpretation should be approached with caution, as differences in treatment strategies, disease duration, local interventions, and other disease characteristics may also account for the observed association. Other US findings, e.g. erosions, enthesitis, bursitis, dactylitis, were not predictive. Conversely, in a prospective study of 107 patients analyzing drug persistence in PsA, Gutierrez et al. reported that a higher baseline burden of synovitis, peritenonitis, and enthesitis was associated with a greater short-term treatment response, whereas a higher burden of bone erosions correlated with earlier drug discontinuation (39). These results suggest differences between predictors of short-term treatment response and predictors of D2T disease. This distinction is clinically relevant, as factors associated with early treatment response may not necessarily predict the long-term trajectory toward a difficult-to-treat phenotype, which likely reflects cumulative inflammatory burden, comorbidities, and therapeutic sequencing over time.

To the best of our knowledge, this is the first real-world study analyzing independent US predictors of D2T PsA, as existing studies have focused primarily on clinical and demographic predictors (2, 9–13). While US has been mainly applied to diagnosis and treatment monitoring so far (40, 41) our findings support its use in prognostic stratification of refractory disease. Importantly, GLOESS and DAPSA scores did not show any correlations, consistent with previous data reporting no (42) or weak-to-moderate correlations (43) between DAPSA and gray-scale synovitis or power Doppler signals. This may suggest that GLOESS and DAPSA scores reflect different dimensions of disease. Indeed, DAPSA can be influenced by non-inflammatory pain, such as that associated with fibromyalgia, as well as by the patient global assessment, which includes also skin and axial manifestations, rather than focusing solely on peripheral involvement, as reflected by the GLOESS score (42).

Our study has several limitations, mainly related to its retrospective nature which may entail a bias of treatment allocation, lack of data on skin involvement, and limited number of patients with early overt US abnormalities, warranting further assessments in larger cohorts. Given the limited sample size of some subgroup analyses and the relatively low frequency of several ultrasonographic abnormalities, model instability and overfitting cannot be completely excluded. Furthermore, US assessments were performed at variable time points during follow-up, which may introduce time-related bias, and the lack of formal inter-reader and intra-reader US reliability assessments should be considered when interpreting the relatively small US differences observed between groups. Because the D2T definition adopted reflects a pragmatic adaptation of RA-derived criteria in a retrospective study design, objective inflammatory measures were not uniformly available at the time of D2T classification across the cohort. Consequently, disease activity assessment reflected routine clinical practice and available clinical documentation. Therefore, these findings should be considered exploratory and hypothesis-generating, and warrant validation in independent cohorts. Nevertheless, they provide valuable real-world data on the prediction of an emerging entity such as D2T PsA.

In early 2026, both GRAPPA and EULAR independently proposed consensus definitions for complex-to-manage/difficult-to-manage and treatment-refractory PsA. These definitions include an umbrella category encompassing all patients with treatment-resistant disease, regardless of the presence of objective inflammation, and a more stringent definition restricted to patients with persistent inflammatory activity (3, 4).

The D2T definition adopted in our study aligns with the broader difficult-to-manage construct proposed by EULAR. Importantly, our study captures patients in a phase preceding the full establishment of treatment-refractory disease, thus providing insight into a potentially modifiable “pre-refractory” stage within the broader complex-to-manage construct.

This aspect may also be relevant when interpreting some of the ultrasonographic findings observed in our cohort. Interpreting higher GLOESS scores at the first MTP in patients who subsequently developed D2T PsA should be approached with caution. This joint is frequently affected by other conditions, such as gout (44) and osteoarthritis (45), which may coexist with PsA. These comorbidities may contribute to the persistence of symptoms through non-inflammatory pain mechanisms. Therefore, a potential confounding effect cannot be completely ruled out and warrants further investigation.

In this retrospective real-world exploratory cohort, a diagnostic delay of at least one year was independently associated with subsequent D2T PsA classification, particularly in stratified analyses of peripheral and axial disease. US findings at the selected index examination, especially higher first MTP GLOESS, were associated with D2T classification, whereas wrist GLOESS showed an inverse association. These findings require prospective external validation using standardized US timing and current consensus definitions of difficult-to-manage and treatment-refractory PsA. Our study provides novel evidence that joint US may support early identification of patients at higher risk of developing D2T PsA, potentially enabling earlier and more tailored therapeutic strategies.

## Data Availability

The raw data supporting the conclusions of this article will be made available by the authors, without undue reservation.
