# Supplementary material for: Clinical and ultrasonographic features preceding a difficult-to-treat phenotype in psoriatic arthritis: a retrospective real-world exploratory cohort study
Source: Front Immunol. 2026 Aug 26;17:1869052. doi: 10.3389/fimmu.2026.1869052 (PMC13553549; doi:10.3389/fimmu.2026.1869052)
Supplement: Supplementary file 1 [file SupplementaryFile1.docx]

**Supplementary figure 1. Flow diagram of patient selection and study cohort derivation.**

Flow diagram of patient screening, eligibility assessment, and inclusion in the clinical and ultrasonographic analyses. Of 178 patients with seronegative spondyloarthritis screened, 152 fulfilled CASPAR criteria for PsA and were included in the clinical cohort. Among them, 117 had an available ultrasound examination and were included in the ultrasonographic analysis (55 D2T PsA and 62 non-D2T PsA).

CASPAR ClASsification criteria for Psoriatic ARthritis; PsA Psoriatic arthritis; SpA spondyloarthropaties; US, ultrasound.

**Supplementary table 1. Overall medication burden.**

PsA, psoriatic arthritis; D2T, difficult-to-treat; Non-D2T, non difficult-to-treat; SD, standard deviation; b/tsDMARDs, biological/targeted synthetic disease-modifying anti-rheumatic drugs; csDMARDs, conventional synthetic disease-modifying antirheumatic drugs; TNFi, Tumor Necrosis Factor inhibitor; il-17i, interleukin 17 inhibitor; IL-12/23i, interleukin 12/23 inhibitor; JAKi, Janus Kinase inhibitor. Rates are calculated on D2T and non-D2T populations.

| **Medication** | **D2T PsA**  **N=81** | **Non-D2T PSA**  **N=71** | **p-value** |
| --- | --- | --- | --- |
| Overall b/tsDMARDs, median (IQR) | 2 (2-3) | 1 (0-1) | <0.001 |
| TNFi, n (%) | 74 (91.3) | 38 (53.5) | <0.001 |
| IL-17i, n (%) | 76 (93.8) | 10 (14.1) | <0.001 |
| IL-12/23i, n (%) | 5 (6.2) | 0 | 0.059 |
| JAKi, n (%) | 22 (27.2) | 2 (2.8) | <0.001 |
| csDMARDs n (%) | 66 (81.5) | 49 (70) | 0.072 |

**Supplementary table 2**. “Reference” ultrasound findings.

PsA, psoriatic arthritis; D2T, difficult-to-treat; Non-D2T, non difficult-to-treat; MCP, metacarpophalangeal joints; PIP, proximal interphalangeal joints, MTP, metatarsophalangeal joints.

| **Ultrasound findings** | **D2T**  **N=55** | **Non-D2T**  **N=62** | **p value** |
| --- | --- | --- | --- |
| Synovitis n (%)   - Wrists - MCP - **PIP** - MTP - Ankles - Knees | 27 (49)  14 (25.4)  10 (18.1)  6 (10.9)  8 (14.5)  5 (9.1)  2 (3.6) | 30 (48.4)  15 (24.2)  11 (17.7)  1 (1.6)  5 (8.1)  7 (11.3)  4 (6.4) | 0.124  0.99  0.451  0.045  0.224  0.221  0.554 |
| Tenosynovitis n (%)   - Wrist extensors - Wrist flexors - Ankles | 15 (27.2)  6 (10.9)  8 (14.5)  3 (5.4) | 10 (16.1)  2 (3.2)  5 (8.1)  4 (6.4) | 0.121  0.101  0.207  0.567 |
| Erosions n (%) | 2 (3.6) | 3 (4.8) | 0.557 |
| Bursitis n (%) | 8 (14.5) | 10 (16.1) | 0.222 |
| Enthesitis n (%) | 1 (1.8) | 4 (6.4) | 0.509 |
| Dactylitis n (%) | 0 | 1 (1.6) | 0.530 |

**Supplementary Table 3**. Correlation with Disease Activity Index for Psoriatic Arthritis (DAPSA) (Spearman’s rho, p value).

GLOESS, Global OMERACT/EULAR Ultrasound Synovitis Score; MCP, metacarpophalangeal joints; PIP, proximal interphalangeal joints, MTP, metatarsophalangeal joints.

| Ultrasound variable | ***r*** | ***p value*** |
| --- | --- | --- |
| *Mean PIP GLOESS* | *-0.07* | *0.751* |
| *Mean MCP GLOESS* | *0.172* | *0.432* |
| *Mean wrists GLOESS* | *-0.41* | *0.854* |
| *Mean ankles GLOESS* | *0.312* | *0.147* |
| *Mean knees GLOESS* | *-0.145* | *0.509* |
| *Mean MTP GLOESS* | *0.249* | *0.251* |
